# Exploring Competency Development Through Simulation-Based Preclinical Training in Veterinary Education

**DOI:** 10.3390/vetsci13030260

**Published:** 2026-03-11

**Authors:** Paz Galarza-Alvarado, Diana Patricia Moya-Loaiza, Fernando Ramonet, Jhonatan Heriberto Vázquez-Albornoz, Freddy Patricio Moncayo-Matute

**Affiliations:** 1 Polivet Veterinary Clinical Department, Universidad Politécnica Salesiana (UPS), Sede Cuenca, Azuay, Cuenca 010102, Ecuador; mgalarza@ups.edu.ec; 2 Research Group on Soft Leadership Skills Towards Sustainable Development (GI-HABLIDES), Universidad Politécnica Salesiana (UPS), Sede Cuenca, Azuay, Cuenca 010102, Ecuador; dmoyal@ups.edu.ec; 3 New Materials and Transformation Processes Research Group (GIMAT), Department of Mechanical Engineering, Universidad Politécnica Salesiana (UPS), Sede Cuenca, Azuay, Cuenca 010102, Ecuador; 4 Institute of Physical and Information Technologies “Leonardo Torres Quevedo” (ITEFI), Spanish National Research Council (CSIC), C/Serrano 144, 28006 Madrid, Spain; 5 Plastic and Reconstructive Surgery Department, Hospital del Río, Cuenca 010109, Ecuador; jhvaplastic@gmail.com

**Keywords:** veterinary training, additive manufacturing, education, teaching, acquired skill

## Abstract

Strengthening key competencies in veterinary preclinical education, such as anatomical identification, spatial–visual reasoning, and anatomical–surgical understanding, is essential for effective preclinical learning. In this context, veterinary preclinical education is undergoing a transformation process in which traditional theoretical–practical approaches show limitations in responding to current educational demands, making it necessary to adopt innovative strategies based on active learning and simulation. This study presents a simulation-based educational approach designed to support competency development within preclinical veterinary education. Using a reproducible and low-cost workflow applied to a real canine cranial case of extra-genital transmissible venereal tumor (TVCT) with frontal bone invasion, used exclusively as a teaching scenario. Fourteen veterinary medicine students from the same institution participated in two instructional conditions: Group A received traditional theoretical instruction (including cadaveric specimens) without the use of 3D-printed models, while Group B participated in simulation-based training supported by virtual planning and a 3D-printed cranial model. Learning outcomes are assessed through structured observation and descriptive analysis. A Likert-type survey was also used to assess satisfaction and engagement among students who participated in the model-supported training, as well as to map competencies across cognitive, visual-spatial, and anatomical-surgical reasoning domains, with evaluation conducted by veterinarians with clinical and teaching experience. Descriptive observations indicated that students participating in the simulation-based training engaged in three-dimensional anatomical exploration of cranial anatomy and case-based anatomical-surgical discussion. In addition, survey responses from Group B indicated high levels of engagement and interest, as well as high perceived usefulness of the model-supported training experience. These findings suggest that simulation-based educational frameworks may offer a safe, transferable, and pedagogically valuable strategy for competency development within preclinical veterinary education.

## Simple Summary

Developing core competencies such as anatomical understanding, spatial reasoning, and early surgical thinking is essential for veterinary students during preclinical training. Traditional teaching approaches often do not allow students to fully integrate these competencies before entering clinical practice. This study explores a simulation-based preclinical learning approach designed to support competency development in a safe and controlled educational environment. Students engaged in simulation-based training with three-dimensional anatomical content through virtual planning and direct interaction with the cranial model. Within this descriptive educational experience, the simulation-based approach was associated with richer spatial exploration and more applied anatomical–surgical reflection in a safe preclinical environment. These findings suggest that simulation may be a valuable educational strategy for supporting essential preclinical competencies in veterinary education.

## 1. Introduction

Contemporary medical education is at a turning point, where traditional teaching models based primarily on theoretical transmission and limited practical experience are proving insufficient to meet the current demands of students, healthcare systems, and society. Various studies have shown that the isolated acquisition of knowledge does not guarantee the development of effective clinical or surgical skills, especially in complex contexts that require the integration of anatomical knowledge, spatial reasoning, and informed decision-making [1].

In this scenario, competency-based education is a key approach for articulating knowledge, technical skills, clinical reasoning, and professional values, promoting active, contextualized, and transferable learning. However, in preclinical training, particularly in surgical areas, a gap persists between the competency objectives stated in curricula and the actual training opportunities, which underscores the need to incorporate innovative educational strategies based on simulation and emerging technologies [2].

In the field of veterinary medicine, these limitations of the traditional training approach become especially evident in the teaching of anatomically complex pathologies, where the integration of theoretical knowledge, spatial visualization, and clinical reasoning is critical [3]. In particular, understanding cranial tumor lesions is one of the most complex challenges in student training, due to the high anatomical complexity of the skull, the overlapping of bone structures, and the three-dimensional nature of infiltration processes [4]. In the educational context, these difficulties are accentuated by the dependence on traditional teaching methods, such as two-dimensional atlases, static radiological images, and the limited availability of anatomical specimens, which are insufficient to clearly represent the extent, depth, and spatial relationships of pathological cranial lesions [1]. This limitation directly impacts students’ ability to develop sound anatomical-clinical reasoning in real-life scenarios.

Computed tomography (CT) has become established as a fundamental tool for the evaluation of cranial pathologies, allowing detailed characterization of bone structures and soft tissues [5]. However, in the educational field, interpreting two-dimensional tomographic images requires a high level of spatial abstraction that students at the early stages of training do not yet master [6]. The difficulty in integrating axial, sagittal, and coronal sections into a three-dimensional mental representation limits effective understanding of tumor invasion patterns, especially in focal bone lesions or superficial infiltration behavior [7].

Three-dimensional reconstruction based on medical imaging and 3D printing has emerged as a valuable tool to strengthen anatomical teaching and preclinical training in health sciences [4,8,9]. Physical 3D-printed models transform complex digital information into manipulable objects that facilitate tactile exploration, spatial visualization, and morphological comprehension of anatomical structures and pathologies [6,10]. Previous studies report that these models improve active learning, knowledge retention, and spatial analysis abilities, particularly when real clinical cases are integrated as structured learning scenarios [5,11,12,13,14,15,16].

Beyond the difficulties in understanding anatomy, veterinary medical students face significant limitations in developing preclinical surgical abilities, especially with regard to integrating anatomical knowledge with surgical reasoning. Traditional training offers limited opportunities to practice delineating resection areas, theoretical anatomical planning of osteotomies, and anticipating reconstructive scenarios in complex pathologies, due to the scarcity of applicable clinical cases, real anatomical variability, and ethical constraints associated with direct training on patients [3]. As a result, students usually begin their clinical practice with limited preparation to analyze surgical approaches, preserve critical structures, and assess three-dimensional bone defects, which directly affects the confidence, safety, and quality of their initial surgical reasoning.

Canine transmissible venereal tumor (CTVT) is a contagious neoplasm that, although it predominantly affects genital structures, can appear in an extra-genital form in a small percentage of cases [6]. Clinically, genital CTVT typically presents as friable, cauliflower-like masses prone to bleeding and associated with serosanguineous discharge and local inflammation. In extra-genital locations, the clinical presentation may vary depending on the affected tissue and can include progressive mass formation, local tissue displacement, or functional alterations. Cranial manifestations with bone involvement are extremely rare and represent a particularly challenging scenario from both a diagnostic and a training standpoint. The scarcity of documented cases and specific teaching resources makes it difficult to address this topic in the teaching-learning process, limiting students’ exposure to this type of pathology [17].

In this context, the present study aims to examine a simulation-based preclinical training approach focused on the development of core educational competencies in veterinary medical students. Rather than emphasizing technical model-generation processes, the study investigates how structured simulation activities using anatomically accurate representations support three-dimensional anatomical understanding, visual–spatial abilities, and preclinical anatomical–surgical reasoning. A real clinical case is employed exclusively as a didactic scenario, not for therapeutic purposes, to facilitate guided analysis and reasoning within a controlled learning environment. Through this approach, the study seeks to characterize and reinforce key competencies relevant to preclinical veterinary education. By promoting structured simulation-based analysis of real clinical scenarios, this approach also seeks to support the early development of competencies that facilitate students’ progressive transition toward clinical reasoning and future clinical practice readiness.

## 2. Methods

### 2.1. Study Design

The research adopts a descriptive design with an educational focus, based on simulation, to analyze the use of a 3D-printed patient-specific cranial bio-model as a preclinical training tool in veterinary surgical training. The sample consists of 14 students from the Veterinary Medicine program at the Salesian Polytechnic University, Cuenca campus, Ecuador (South America), aged between 22 and 25 years old. The participants were in their fifth and sixth semesters of a nine-semester curriculum. Students from these two consecutive semesters were included because both correspond to the same preclinical stage of the curriculum and share comparable foundational training in anatomy and basic clinical sciences, ensuring a similar baseline level of knowledge for the educational activities.

None of the students had prior experience in simulation-based training using 3D-printed models. However, four participants reported having basic knowledge of surgical planning, although all were considered beginners in the use of virtual surgical planning and simulation with 3D physical models. The students were selected by convenience sampling, based on their availability and willingness to participate in the study. Participants were then assigned to the two groups based on academic scheduling and availability during the teaching activities, ensuring that both groups had comparable training levels. This allocation followed the normal organization of the educational sessions rather than a randomized experimental design, consistent with the exploratory and pilot nature of the study. All activities were carried out exclusively for educational purposes, within a controlled preclinical environment.

### 2.2. Simulation-Based Preclinical Training

The students were divided into two training groups. Group A (n = 7) approached case analysis using traditional teaching methods, utilizing bibliographic resources, two-dimensional anatomical diagrams, and tomographic images without the support of printed bio-models. Group B (n = 7) received simulation-based training, integrating virtual surgical planning and direct manipulation of a 3D-printed cranial model for three-dimensional analysis of the bone defect, and the theoretical delimitation of simulated surgical scenarios.

### 2.3. Learning Outcomes Evaluation

The learning outcomes of the participants were evaluated using a descriptive approach, focused on analyzing preclinical performance in simulation scenarios. The process considered the three-dimensional anatomical understanding of the skull, the spatial analysis of the bone defect, and the anatomical-surgical reasoning applied to simulated surgical planning, as well as the identification of the competencies developed and those required for more complex preclinical surgical scenarios, according to the competency framework proposed in this study.

The educational experience was examined using a descriptive educational approach through structured observation and competency mapping conducted during the development of the training activities. The two instructional conditions were included to characterize different educational dynamics within the same preclinical setting, rather than to support inferential comparison. Accordingly, the study was not intended to test hypotheses, establish statistical differences, or demonstrate the effectiveness or superiority of one instructional strategy over another.

Additionally, a Likert-type survey was applied, adapted from previous references [13], exclusively to Group B, with the objective of gathering students’ self-reported perceptions regarding virtual planning and the use of the 3D-printed cranial model as educational tools, also to assess the level of commitment, understanding, and perceived academic confidence. Consequently, the results derived from this instrument are interpreted as descriptive feedback of the group exposed to simulation-based training, and not as a direct quantitative comparison between groups.

Accordingly, Group A followed the standard instructional approach and was included as a contextual educational reference rather than as a formal comparative arm. The questionnaire was administered only to Group B, as it was specifically designed to assess students’ perceptions of the simulation-based intervention implemented in the simulation-based training condition.

The evaluation was carried out by veterinarians with clinical and teaching experience. They performed the assessment and analyzed the results in a consensual manner, prioritizing coherence between the evaluation criteria and the participants’ preclinical training levels. The observation criteria and competency mapping were previously defined by consensus among the evaluators in order to ensure coherence and descriptive consistency.

### 2.4. Ethical Considerations

The research was conducted in accordance with the ethical principles applicable to educational research involving human participants. Student participation was voluntary, informed consent was obtained, and the confidentiality and exclusive academic use of the collected information were guaranteed.

The animal clinical case used as the basis for generating the cranial bio-model is a retrospective study conducted for diagnostic purposes, without additional intervention or experimental procedures within the framework of this research. According to the Organic Law of Animal Welfare (LOBA), in force in Ecuador, no animal experimentation is permitted; therefore, approval by an animal ethics committee is not required.

### 2.5. Educational Sequence for Model-Supported Simulation

To support a structured preclinical learning experience, the training followed a staged educational sequence that guides students from case interpretation to three-dimensional understanding and simulated planning. Each stage was used to help learners connect clinical findings, imaging information, and anatomical relationships, strengthening spatial reasoning and early anatomical–surgical thinking. The physical model was incorporated as a learning aid to facilitate hands-on exploration and discussion during simulation activities.

**Figure 1:**
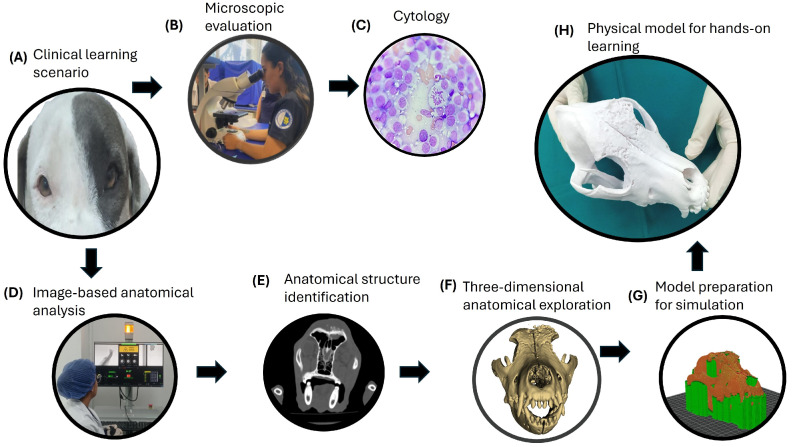
Educational training sequence used for simulation-based preclinical learning. The sequence illustrates the progression of learning activities from case analysis and diagnostic interpretation (**A**–**C**), through image-based anatomical understanding (**D**,**E**), to three-dimensional spatial exploration and simulated anatomical–surgical reasoning using a physical model (**F**–**H**). The technical steps involved are presented as supporting tools for competency development rather than as learning outcomes themselves.

Figure 1 summarizes the training sequence.

### 2.6. Clinical Learning Scenario

In this phase, students are guided to approach the case as a structured learning scenario, prioritizing the interpretation of clinical and imaging information to achieve an integrated understanding of the cranial defect. The objective of this stage is for students to construct an initial mental representation of the anatomical problem, establishing a conceptual basis that supports three-dimensional analysis and subsequent simulation activities.

To support this educational process, a real canine clinical case was incorporated as a didactic resource, providing authentic anatomical and imaging information for preclinical training. The case corresponds to a young adult female dog (Pitbull breed, 1 year and 6 months old, 21.8 kg) presenting a firm, well-defined, painless mass located in the left frontal region of the skull, with progressive growth and no evidence of ulceration or exudate.

From an educational perspective, the lesion—approximately seven centimeters in diameter—was used to challenge students’ spatial interpretation of cranial anatomy and the relationships between osseous structures and surrounding soft tissues.

**Figure 2:**
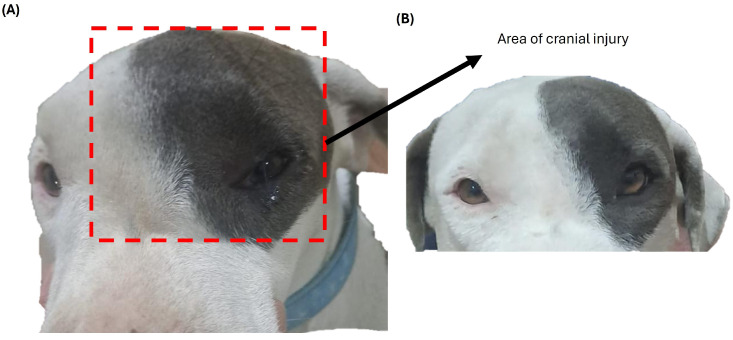
External clinical features of a canine case used for preclinical educational purposes. (**A**) Left-lateral view showing a firm, well-defined mass over the left frontal region, producing mild facial asymmetry. (**B**) Frontal view after cleansing of the affected area, demonstrating superficial soft tissue displacement without ulceration, hemorrhage, or ocular involvement.

The resulting mild facial asymmetry and superficial soft-tissue displacement served as key features for guided anatomical analysis during simulation-based learning (Figure 2A,B).

### 2.7. Cytology as a Training Tool

Cytological analysis was incorporated as a training tool to help students understand the relationship between microscopic findings and the anatomical and imaging alterations observed at the macroscopic level. This stage supports the development of initial anatomical–clinical reasoning by guiding learners to correlate cellular morphology with tissue behavior and structural changes, without implying therapeutic decision-making.

From an educational perspective, cytology reinforces systematic observation, the recognition of basic morphological patterns, and the integration of diagnostic information within a structured learning sequence. These activities prepare students for subsequent three-dimensional anatomical analysis and simulation-based exploration in later phases of the training process.

For educational purposes only, fine needle aspiration cytology (FNAC) was performed to provide representative cytological material for guided microscopic interpretation. During this activity, students were introduced to key cytomorphological features of neoplastic processes, including round cell populations, cytoplasmic vacuolization, nuclear variation, and mitotic activity, as a means of strengthening pattern recognition and diagnostic reasoning skills [18].

**Figure 3:**
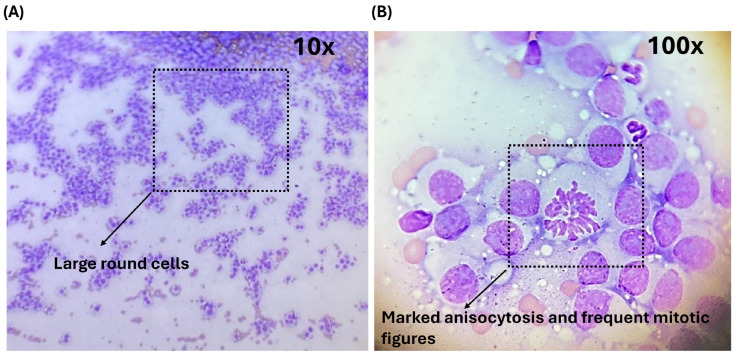
Cytological images used for guided microscopic interpretation during preclinical training. (**A**) Diff-Quick-stained smear at low magnification (10×), showing large round cells with basophilic cytoplasm and cytoplasmic vacuoles. (**B**) Higher-magnification view (100×) demonstrating nuclear variation and frequent mitotic figures. These features were used to support pattern recognition and diagnostic reasoning in a simulation-based educational setting.

The observed cytological features were consistent with those typically described for CTVT in its extragenital cranial presentation, serving as a reference framework for discussion and comparative analysis rather than as a focus on clinical diagnosis. Representative cytological fields illustrating these features are shown in Figure 3A,B and were used to support guided learning and discussion.

### 2.8. Clinical Preparation of the Patient

Fine needle aspiration cytology was performed following World Small Animal Veterinary Association (WSAVA) guidelines. Due to the osseous involvement, an 18-gauge needle attached to a 10-mL syringe was used to access osteolytic areas, with multiple gentle passes to optimize cellular yield. Smears were prepared immediately using a squash technique, air-dried, and stained with Wright stain. Local analgesia was provided with 2% lidocaine infiltration, followed by postoperative meloxicam.

Cranial CT was acquired with the patient in sternal recumbency under clinical supervision. Sedation with tiletamine–zolazepam (Zoletil^®^) was administered to ensure adequate immobilization, and physiological parameters were monitored throughout the procedure. Images were obtained using a standard helical cranial CT protocol with thin slices and bone reconstruction, without intravenous contrast, as the study focused on structural bone assessment. The patient recovered uneventfully under post-procedural observation.

### 2.9. Imaging as a Preclinical Learning Tool

In the context of preclinical training, imaging was incorporated as a learning tool to strengthen anatomical–spatial reasoning and to support the interpretation of relationships between bone structures and surrounding soft tissues. This stage allows students to conceptually define lesions, integrate imaging findings with previous clinical and cytological information, and transition from diagnostic observation to three-dimensional anatomical understanding and simulated surgical planning.

As part of this educational activity, a high-resolution CT study of the cranial region was used to provide detailed anatomical information for guided analysis. Rather than focusing on the technical execution of image acquisition, the emphasis of this stage was placed on student interaction with the images to identify anatomical landmarks, recognize pathological alterations, and interpret spatial relationships relevant to preclinical surgical reasoning [19].

CT images were subsequently processed to obtain three-dimensional representations that facilitated visual–spatial exploration and discussion. Through guided segmentation and reconstruction activities, students were able to delineate cranial structures and appreciate the extent and localization of the lesion, supporting the development of spatial awareness and anatomical reasoning. These steps were implemented as part of the simulation-based learning sequence and not as an isolated technical workflow [20,21].

From an educational perspective, the tomographic findings—characterized by a focal osteolytic defect in the left frontal bone, cortical irregularities, and associated soft tissue changes—served as reference features for structured analysis and discussion. The absence of intracranial, orbital, or nasal cavity involvement was used to guide students in distinguishing localized cranial processes from more extensive pathological conditions. These imaging characteristics were integrated with cytological observations to reinforce multidisciplinary reasoning during preclinical training.

**Figure 4:**
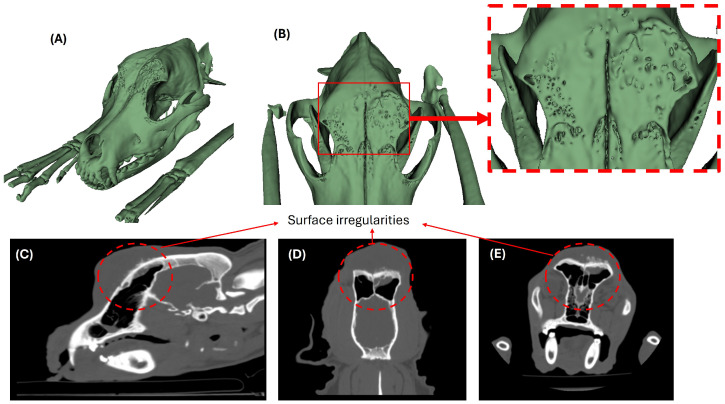
Imaging-based learning sequence used during simulation-based preclinical training. (**A**,**B**) Three-dimensional reconstructions employed to facilitate spatial understanding of cranial anatomy and lesion localization. (**C**–**E**) Multi-planar CT images used for guided anatomical interpretation of bone and soft tissue relationships during preclinical training activities.

Figure 4 illustrates the imaging-based learning sequence, including three-dimensional reconstructions and multi-planar tomographic views, which were used to support guided anatomical interpretation and simulation-based exploration.

### 2.10. Anatomical 3D Visualization

Anatomical three-dimensional visualization is a key stage in supporting students’ understanding of how medical imaging data can be transformed into coherent and interpretable anatomical representations for educational purposes. This stage strengthens the integration of anatomy, imaging, and spatial reasoning, allowing students to better appreciate the structural continuity of the skull and the relationship between the bone defect and surrounding tissues within a simulation-based learning context.

From a learning perspective, the digital model derived from tomographic segmentation was refined to facilitate clear and accurate anatomical visualization during preclinical training activities. Rather than emphasizing the technical aspects of model preparation, this process was oriented toward ensuring that students could clearly identify relevant anatomical landmarks, lesion boundaries, and spatial relationships during guided exploration and discussion.

The refinement of the three-dimensional model included the removal of non-anatomical elements and the correction of minor geometric inconsistencies that could interfere with visual interpretation. These steps were implemented to preserve anatomical fidelity and to enhance the educational value of the model as a visualization and simulation resource, without altering the original anatomical morphology.

Through interaction with the refined three-dimensional model, students were able to explore cranial anatomy from multiple perspectives, supporting the development of visual–spatial skills and a deeper understanding of three-dimensional anatomy. This visualization stage served as a bridge between image-based analysis and hands-on simulation, reinforcing anatomical–surgical reasoning in a preclinical learning environment.

**Figure 5:**
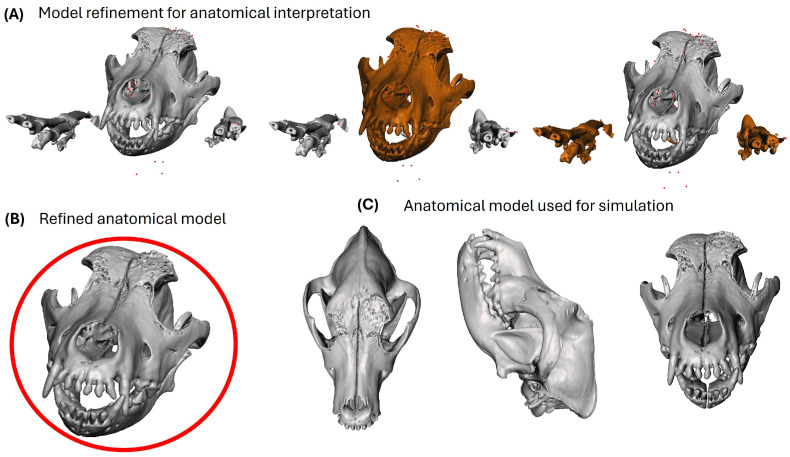
Three-dimensional anatomical visualization used during simulation-based preclinical training. (**A**) Initial refinement of the segmented model to improve anatomical clarity. (**B**) Refined cranial model used for guided anatomical exploration. (**C**) Multi-view representation of the final model supporting spatial understanding in preclinical training.

Figure 5 illustrates the progressive stages of anatomical visualization used during training, from initial model refinement to the final three-dimensional representation applied in simulation-based activities.

### 2.11. Fabrication of the Physical Model for Preclinical Training

The anatomical cranial model was fabricated to provide students with a tangible learning resource that supports hands-on exploration during simulation-based preclinical training. The physical model was generated from the three-dimensional digital reconstruction obtained after tomographic segmentation, ensuring anatomical correspondence with the original imaging data.

From an educational perspective, a low-cost additive manufacturing approach was selected to facilitate reproducibility and accessibility within academic training contexts. The model was manufactured using fused deposition modeling (FDM) technology and polylactic acid (PLA), a thermoplastic material widely used in educational environments due to its dimensional stability, availability, and affordability. The use of low-cost materials was intended to support the potential scalability of this approach within veterinary education programs.

During fabrication, automated support structures were generated to preserve anatomical fidelity in regions with overhangs and internal cavities. These supports were used exclusively to ensure structural stability and surface integrity of the model, without altering the anatomical characteristics of the regions of interest. The orientation of the model during printing was selected to optimize the clarity of key anatomical features relevant to spatial understanding and simulation activities.

Once manufactured, the physical cranial model was incorporated as a hands-on learning tool within the simulation-based preclinical training sequence. The printed model enabled students to interact directly with three-dimensional anatomical structures, reinforcing spatial reasoning, anatomical comprehension, and preclinical anatomical–surgical thinking in a safe and controlled educational environment.

**Figure 6:**
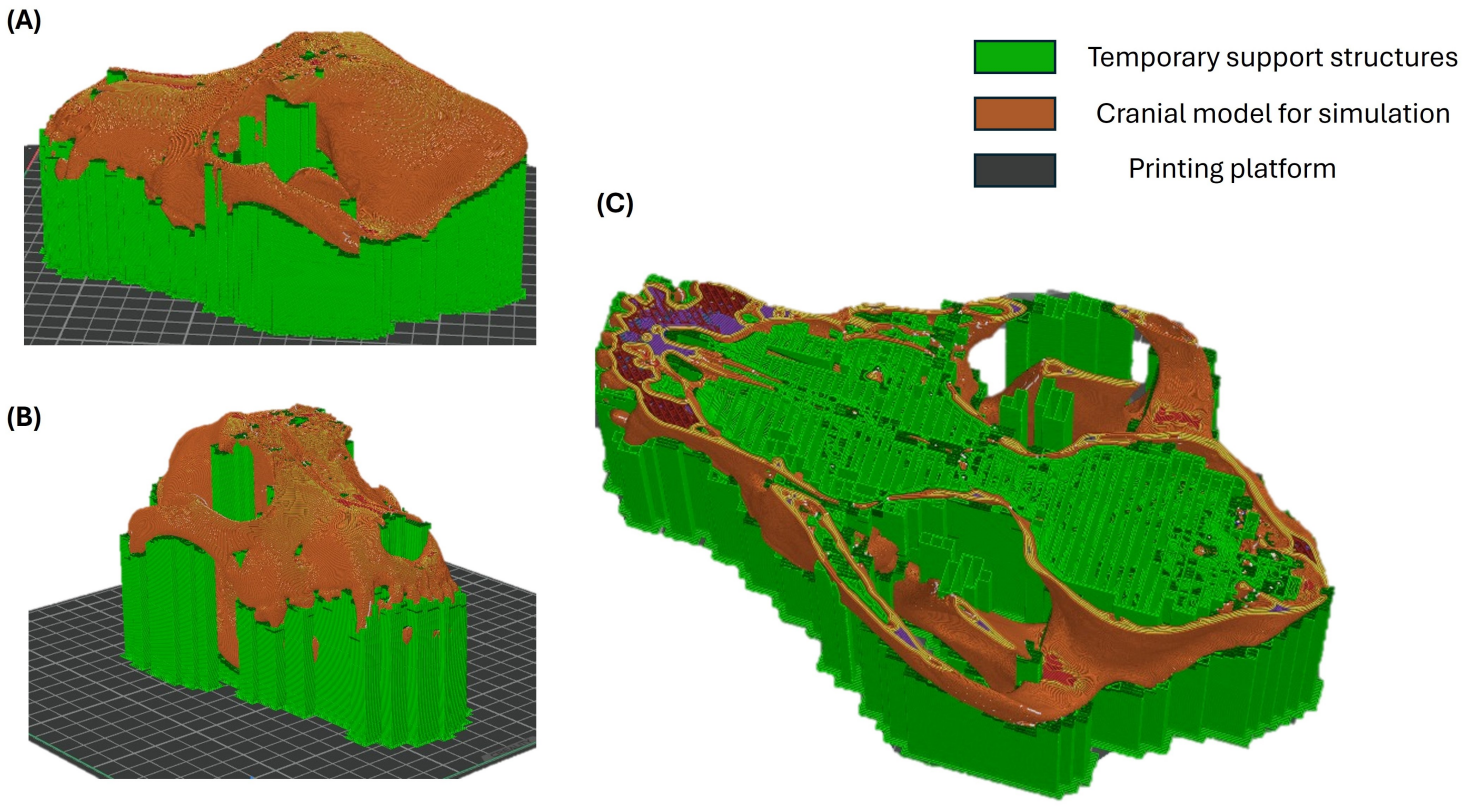
Preparation of the physical cranial model used for simulation-based preclinical training. (**A**–**C**) Digital preparation stages of the cranial model illustrating the generation of temporary support structures to preserve anatomical integrity during fabrication. These steps were implemented to ensure structural stability and fidelity of the model prior to its use as a hands-on educational resource in preclinical simulation activities.

Figure 6 illustrates the fabrication stages of the cranial model as part of the educational workflow, highlighting how the physical model was prepared for use in simulation-based training.

Once prepared, the physical cranial model was incorporated as a hands-on learning resource within the simulation-based preclinical training sequence, serving as the central interface for the structured educational stages outlined in Table 1.

### 2.12. Simulation-Based Hands-On Session

**Figure 7:**
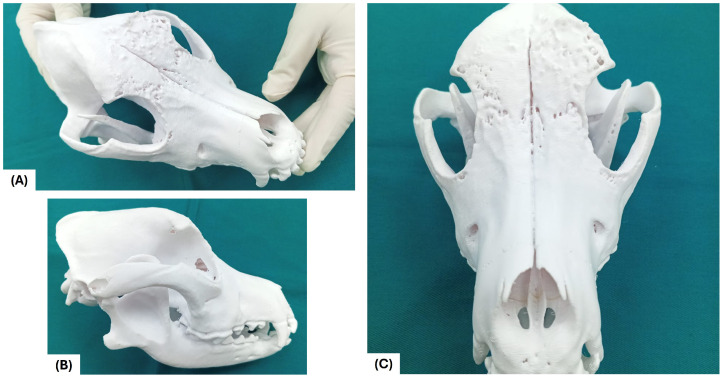
Cranial model for anatomical visualization and preclinical training. (**A**) Dorsolateral view illustrating a focal frontal osteolytic defect with preserved midline anatomy. (**B**) Left lateral view showing irregular cortical margins and localized bone loss without orbital involvement. (**C**) Frontal view demonstrating intact nasal cavity boundaries and overall cranial symmetry.

Through three-dimensional visualization of the extent, geometry, and depth of the frontal osteolytic defect associated with extra-genital CTVT, the model facilitates spatial understanding of left frontal cortical bone loss and its relationship to critical anatomical structures, without evidence of involvement of the nasal cavity, orbit, or cranial midline (Figure 7).

This approach may help address some limitations of conventional two-dimensional image interpretation, providing a more tangible and structured understanding of cranial anatomy in a safe educational setting.

The analysis of the bio-model from dorsolateral (Figure 7A), left lateral (Figure 7B), and frontal (Figure 7C) views allows training in the identification of irregular bone margins, the assessment of the depth of the defect, and the appreciation of overall cranial symmetry.

These observations may be relevant for the development of three-dimensional visualization skills and anatomical reasoning, particularly in complex or rare cranial pathologies, where spatial understanding is a key component of preclinical training.

**Figure 8:**
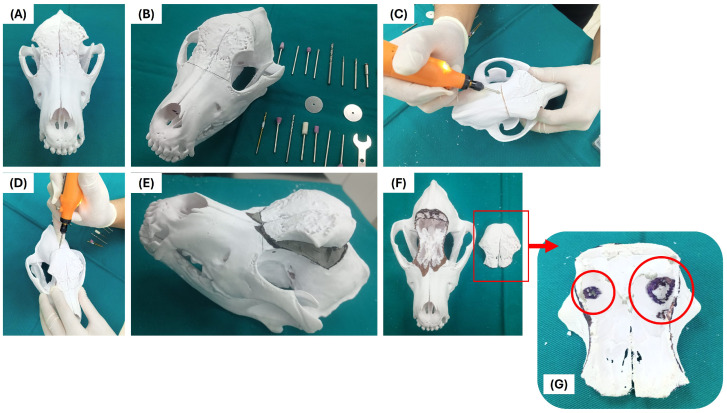
Simulation-based surgical training using the cranial model. (**A**,**B**) Delineation of hypothetical osteotomy lines on the bio-model for training purposes, respecting anatomical landmarks. (**C**,**D**) Stepwise simulation of bone removal to explore theoretical exposure of the frontal defect while preserving critical structures, such as the orbit. (**E**–**G**) Evaluation of the resulting simulated defect.

Subsequently, the bio-model is used to simulate hypothetical surgical scenarios, aimed at training in the delimitation of osteotomy lines and bone resection trajectories (Figure 8). This allows students to analyze which bone areas can theoretically be resected, which should be preserved, and which regions would require eventual reconstruction, respecting critical structures such as the orbit, the zygomatic arch, and the limits of the nasal cavity (Figure 8A,B).

The exercise focuses on acquiring competence related to theoretical anatomical planning and simulated anatomical-surgical decision-making, without the model being used for real clinical decision-making.

The training also included the evaluation of the resulting defect after simulated resection and the discussion of possible reconstructive strategies, such as the hypothetical use of autologous bone grafts or inert biomaterials, with the aim of restoring cranial convexity and protecting adjacent cavities (Figure 8C–F). This allows for the exploration of multiple reconstructive scenarios in a controlled environment, promoting case-based learning and critical reflection without risk to the patient.

This approach highlights the potential educational value of the bio-model as a reproducible resource for preclinical surgical training, in a context that supports the exploration of hypothetical surgical strategies and guided reflection before actual clinical contact.

The feedback session allowed the consolidation of learning derived from the simulation experience, promoting critical reflection on the use of virtual planning and the cranial models as educational tools.

Within this descriptive educational framework, Table 2 provides an overview of the two instructional conditions (Group A and Group B) and summarizes the main pedagogical elements observed during the activities. This table is intended to document the characteristics of each instructional condition and the types of student interactions supported by each approach, rather than to establish inferential comparisons between groups.

Table 3 shows the items in the questionnaire used to analyze the perception of Group B after the training.

## 3. Results

### 3.1. Learning Outcomes

The results are organized to describe the main learning outcomes observed across the two instructional conditions. The learning outcomes are presented based on a descriptive analysis of students’ educational performance, taking into account structured observation, competency mapping, and feedback collected through the satisfaction and engagement survey administered to Group B.

From a descriptive perspective, observation and competency mapping were used to characterize how students approached the anatomical and spatial analysis of the simulated scenarios. In the simulation-based training condition (Group B), students interacted directly with the three-dimensional model and participated in guided anatomical–surgical discussions supported by virtual planning and the 3D-printed cranial model. In the standard instructional condition (Group A), activities relied primarily on conventional resources, including bibliographic material, anatomical diagrams, and tomographic images, without manipulation of a physical model.

Across the simulation-based sessions, students engaged in hands-on three-dimensional interaction and case-based anatomical–surgical reflection within a controlled preclinical setting. Regarding anatomical understanding and spatial analysis of bone defects, observations in the simulation-based condition frequently included identification of complex anatomical relationships and integration of imaging with three-dimensional representation. In the standard instructional condition, learning activities remained centered on image-based and two-dimensional interpretation.

The satisfaction and commitment questionnaire, administered exclusively to students in Group B (see Table 4), explored participants’ self-reported perceptions regarding the use of 3D-printed cranial models and virtual planning as educational tools. Survey responses indicated high levels of engagement and interest, as well as high perceived usefulness of the model-supported training experience and perceived academic confidence during the simulation-based training.

Participants also highlighted the usefulness of the model for visualizing complex anatomical relationships, reinforcing key concepts, and facilitating the repetition of analysis and planning exercises in a controlled environment.

Although this study represents an initial approach with a limited number of students, the descriptive findings suggest that simulation using cranial models may support preclinical surgical training by enabling structured three-dimensional exploration and guided case-based discussion within a safe educational environment.

### 3.2. Implications for Curriculum Design and Competency Development

In order to systematise the educational contribution of this study from an educational perspective, the competencies observed and perceived as developed during simulation-based preclinical training are identified, as well as those additional competencies that would be required to address more complex cranial surgical scenarios, according to the literature [22,23]. This differentiation allows us to contextualize the educational scope of the work and guide curricular reflection on the use of 3D models in preclinical surgical training.

In Table 5, a descriptive mapping of competencies is presented, organized into cognitive, visual and spatial, psychomotor, surgical reasoning, reconstructive, and professional-ethical domains. This mapping is not proposed as a normative standard or a prescriptive framework for curriculum progression; instead, it serves as a guiding input to visualize the competencies that may be developed through simulation-based training with cranial bio-models derived from CT.

From a curricular perspective, the study’s findings suggest that the progressive incorporation of simulation strategies could contribute to strengthening the integration between applied anatomy, three-dimensional spatial analysis, and anatomical-surgical reasoning in the early stages of training. In this regard, preclinical training in controlled environments offers students structured opportunities to explore complex scenarios without risk to patients, facilitating the transition from theoretical learning to initial clinical understanding.

Furthermore, the methodological workflow, designed based on CT, three-dimensional reconstruction, and low-cost 3D printing, suggests potential institutional transferability, as it is accessible, reproducible, and adaptable to different subjects in the veterinary curriculum, such as Applied Anatomy, Diagnostic Imaging, and Preclinical Surgical Training.

The explicit identification of competencies associated with this type of training can serve as a basis for curriculum improvement processes, aligned with contemporary approaches to competency-based education, without overstating the scope of this study.

## 4. Discussion

This pilot educational experience explored the use of a patient-specific 3D-printed cranial model, derived from a real canine clinical case and used exclusively as a teaching scenario, as a support tool for preclinical veterinary training. Within this context, the descriptive findings suggest that model-supported simulation may offer educational value for strengthening anatomical understanding, spatial reasoning, and applied anatomical–surgical discussion in a controlled preclinical environment [28].

Anatomical three-dimensionality and physical interaction with printed models were observed to facilitate spatial understanding, anatomical orientation, and procedural reasoning, which are foundational competencies for subsequent clinical and surgical training. In this regard, the integration of simulation-based learning with anatomically accurate three-dimensional models may not only support competency development in preclinical stages but also contribute to strengthening students’ preparedness for subsequent decision-making processes through repeated observation and reflective guided analysis [15].

Unlike conventional two-dimensional imaging or exclusively theoretical instruction, hands-on interaction with the three-dimensional model in this pilot experience was associated with opportunities for students to engage with complex anatomical structures in a safe, controlled, and repeatable educational setting. These characteristics may support cognitive engagement and experiential learning. The use of real-case derived anatomical models may be particularly relevant in educational contexts in which clinical meaningfulness enhances student motivation and supports the connection between theoretical content and applied reasoning [29].

These findings are consistent with educational frameworks that emphasize the early development of integrative cognitive skills alongside technical knowledge, simulation-based pedagogies, and experiential learning in preclinical education. Although much of the existing literature has focused on communication and clinical interaction skills, the pedagogical principles underlying simulation-based learning may also be applicable to preclinical surgical education, particularly for supporting structured anatomical interpretation and case-based reasoning [30].

A key contribution of this study lies in its contextual relevance as a veterinary educational experience developed in Latin America. While educational research on simulation remains concentrated in North America and Europe, this work contributes to expanding the implementation of low-cost and reproducible educational resources in a regional veterinary training context. The proposed methodological workflow, based on CT, three-dimensional reconstruction, and low-cost 3D printing, suggests potential instructional transferability, as it may be adapted to different subjects such as Applied Anatomy or surgical training in settings with limited resources.

As an exploratory pilot study, this work was not designed to measure clinical performance outcomes, but rather to examine how a model-supported educational experience may function in a preclinical teaching framework. As a pilot educational experience, the findings provide a basis for future studies with larger cohorts, more structured assessment instruments, and broader educational contexts.

Overall, simulation-based training using 3D-printed anatomical models may represent a pedagogically valuable strategy for supporting active learning, spatial understanding, and case-based reflection within preclinical veterinary education. However, additional studies are needed to determine how these educational experiences may relate to subsequent performance in clinical or surgical training contexts across different institutional and geographical settings.

## 5. Research Limitations

The study is constrained by its limited sample size, a factor that restricts the generalizability of the results to broader educational contexts. The analysis is based on a single clinical case, used exclusively as a teaching scenario, and is mainly descriptive and perception-based in nature, without standardized objective metrics or longitudinal follow-up to evaluate retention or its transfer to real clinical contexts. Because the questionnaire was administered only to Group B, survey items framed relative to “traditional” instruction reflect participants’ self-reported perceptions and should not be interpreted as evidence of comparative effectiveness between groups.

Furthermore, neither the exposure time nor the possible novelty effect associated with the incorporation of integrated technologies was independently controlled. Consequently, the findings reflect the impact of the integrated teaching approach as a whole, which combines methodological flow, virtual planning, and simulation with cranial models, rather than the isolated effect of a single component, such as the physical model.

In addition, the study is not designed to establish causal relationships or to determine whether the observed changes in the learning experience were directly attributable to the physical model itself rather than to other factors such as group dynamics and teaching strategies. Additionally, the fidelity of the biomodels used, which can influence perceived anatomical realism, depends on the quality of the tomographic acquisition, segmentation accuracy, and the digital reconstruction process.

Future studies with larger samples, multiple clinical cases, validated instruments, and more robust comparative designs are needed to further investigate the educational impact of simulation-based training and assess its applicability in different surgical training programs.

## 6. Conclusions

This study presents a simulation-based educational approach that integrates computed tomography, three-dimensional reconstruction, and 3D printing to support preclinical surgical training in veterinary medicine, using a real case exclusively as a teaching scenario. The descriptive findings suggest that this approach may support learning through three-dimensional anatomical understanding and spatial analysis in controlled and safe educational environments. Within this pilot educational experience, the model-supported training condition was associated with opportunities for contextualized preclinical discussion and applied anatomical–surgical reasoning.

Survey responses from students participating in the simulation-based training indicated high levels of engagement and interest, as well as positive perceived usefulness of the 3D-printed models as educational resources for supporting active learning. Likewise, the proposed methodological workflow may favor the progressive integration of clinical imaging and anatomical information, thereby contributing to a more contextualized approach to preclinical anatomical–surgical reasoning.

From an educational perspective, the study outlines a replicable training framework that may be adapted to different educational subjects and contexts, including anatomy, surgical training, minimally invasive procedural practice, or specialty-focused learning (e.g., cardiology), without replacing clinical training. Although the results are based on a limited sample and descriptive instruments, this pilot educational experience suggests that 3D-printed models, as part of a simulation-based training strategy, may offer value for future educational research with more robust designs.

## Figures and Tables

**Table 1:** Stages of the simulation-based preclinical training program.

Stage of the Training Program	Overview
Introductory session	The initial stage aimed at contextualizing students in simulation-based preclinical learning, introducing the educational objectives of the training program, and its role in competency development. This phase focuses on reinforcing core anatomical concepts, basic interpretation of clinical findings, and the integration of imaging information to support three-dimensional anatomical understanding. Digital tools and three-dimensional representations are introduced as supportive resources to facilitate visualization and conceptual exploration of cranial anatomy and structural alterations.
Simulation-based practical session (hands-on)	This stage focuses on direct interaction with the 3D-printed cranial model, designed for three-dimensional exploration, spatial analysis of the bone defect, and the development of preclinical competence through simulated surgical scenarios. This phase allows for the progressive integration of anatomical knowledge with applied reasoning in a controlled environment.
Feedback and debriefing session	The final stage focuses on critical reflection and consolidation of learning. It includes guided discussion of the simulation experience, analysis of decisions made during practice, and linking the activities performed with the competence developed, fostering meaningful and progressive learning.

**Table 2:** Descriptive overview of the two instructional conditions.

Evaluated Aspect	Group A	Group B
Number of students	7	7
Instruction	Theoretical analysis using conventional resources	Simulation-based structured program
Analysis of medical examinations (clinical and imaging)	Included (conventional clinical analysis)	Included (clinical analysis prior to simulation)
Introductory session	Conventional theoretical introduction	Structured introduction with image-based learning and spatial reasoning
Use of virtual planning	Not included	Included
Use of 3D-printed cranial bio-model	Not included	Included
Three-dimensional interaction	Limited to two-dimensional images	Direct manipulation of the printed physical model
Analysis of the bone defect	Conceptual and two-dimensional	Three-dimensional and spatial
Development of visual and spatial competence	Basic	Reinforced
Preclinical anatomical-surgical reasoning	Theorist	Applied in simulated scenarios
Anatomy-imaging integration	Partial	Improved
Feedback and debriefing session	Unstructured	Included and guided
Perceived anatomical understanding	Standard	Improved
Perceived academic confidence	Standard	Increased

**Table 3:** Survey items on perception given to Group B.

Question	Answers
Rate your overall level of engagement during the educational session	1—Nothing committed
	2—Not very committed
	3—Moderately committed
	4—Committed
	5—Very Committed
How would you rate your interest in the learning materials?	1—Very low
	2—Low
	3—Moderate
	4—High
	5—Very High
Does the methodology used facilitate anatomical identification with the help of the 3D model?	1—Yes, significantly
	2—Yes, to some extent
	3—Not really
	4—No, not at all
How would you rate the clarity and visual appeal of the educational resource used?	1—Very poor
	2—Poor
	3—Good
	4—Very good
	5—Excellent
Compared to the traditional method, do you consider this methodology to make learning more interactive?	1—Yes, significantly more interactive
	2—Yes, something more interactive
	3—No, a similar level of interaction
	4—No, less interactive
Does the methodology used improve your understanding compared to the traditional method?	1—Yes, significantly
	2—Yes, to some extent
	3—Not really
	4—No, not at all
How confident do you feel about applying the knowledge you have acquired in preclinical settings?	1—Very confident
	2—Confident
	3—Moderately secure
	4—Not at all secure
Does the methodology facilitate your ability to understand complex spatial concepts and relationships?	1—Yes, significantly
	2—Yes, to some extent
	3—Not really
	4—No, not at all
Grade the perceived improvement in your knowledge and skills after the educational session	1—Without improvement
	2—Slight improvement
	3—Moderate improvement
	4—Significant improvement
	5—Very significant improvement
In general terms, is the learning experience better than with the traditional approach?	1—Indeed, significantly superior
	2—Yes, something better
	3—Similar
	4—No, it was worse

**Table 4:** Group B’s opinion on the educational experience with simulation using a cranial model.

Question	1	2	3	4	5	6	7
Rate your overall level of engagement during the educational session	Very Committed	Very Committed	Very Committed	Committed	Committed	Very Committed	Committed
How would you rate your interest in the learning materials?	High	Very High	High	High	Very High	High	Very High
Does the methodology used facilitate anatomical identification with the help of the 3D model?	Yes, to some extent	Yes, to some extent	Yes, to some extent	Yes, significantly	Yes, to some extent	Yes, significantly	Yes, to some extent
How would you rate the clarity and visual appeal of the educational resource used?	Very good	Very good	Good	Excellent	Very good	Excellent	Excellent
Compared to the traditional method, do you consider this methodology to make learning more interactive?	Yes, significantly more interactive	Yes, something more interactive	Yes, something more interactive	Yes, significantly more interactive	Yes, something more interactive	Yes, something more interactive	Yes, significantly more interactive
Does the methodology used improve your understanding compared to the traditional method?	Yes, significantly	Yes, significantly	Yes, significantly	Yes, significantly	Yes, significantly	Yes, to some extent	Yes, to some extent
How confident do you feel about applying the knowledge you have acquired in preclinical settings?	Very confident	Very confident	Moderately secure	Very confident	Very confident	Moderately secure	Very confident
Does the methodology facilitate your ability to understand complex spatial concepts and relationships?	Yes, significantly	Yes, to some extent	Yes, significantly	Yes, significantly	Yes, significantly	Yes, to some extent	Yes, significantly
Grade the perceived improvement in your knowledge and skills after the educational session	Moderate improvement	Significant improvement	Moderate improvement	Significant improvement	Moderate improvement	Moderate improvement	Significant improvement
In general terms, is the learning experience better than with the traditional approach?	Indeed, significantly superior	Indeed, significantly superior	Indeed, significantly superior	Indeed, significantly superior	Indeed, significantly superior	Yes, something better	Indeed, significantly superior

**Table 5:** Descriptive mapping of competencies promoted during simulation-based preclinical training and competencies required for more complex surgical scenarios.

Area of Competence	Competencies Promoted During Preclinical Training	Competencies Required for More Complex Surgical Scenarios
**Cognitive (anatomical-clinical)**	Three-dimensional understanding of cranial anatomy, analysis of the extent and depth of tumor invasion, and image-model-bone structure correlation.	Dynamic assessment of oncological margins, integration with cytopathological information, multi-compartmental analysis of the skull/[22].
**Visual-spatial**	Identification of spatial relationships between bone defect, orbit, and nasal cavity; assessment of cranial symmetry and frontal convexity.	Planning complex reconstructions, analysis of extensive post-resection deformities/[24].
**Psychomotor (preclinical)**	Theoretical delineation of osteotomy lines, bone resection simulation, and controlled anatomical manipulation of the bio-model.	Performing osteotomies with real surgical instruments, simulating fixation, and evaluating the mechanical stability of reconstructions/[25].
**Surgical reasoning**	Analysis of hypothetical surgical scenarios, simulated anatomical-surgical decision-making, and theoretical preservation of critical structures.	Real-time intraoperative decision-making, management of simulated complications, dynamic adaptation of the surgical plan/[26].
**Skull reconstruction**	Theoretical discussion of reconstructive strategies (bone grafts, inert biomaterials), morphological assessment of the defect.	Design and validation of customized implants, advanced selection of biomaterials according to mechanical load and biocompatibility criteria/[15].
**Professional and ethical competencies**	Training in a controlled environment, risk-free learning for patients, and critical reflection based on cases.	Interdisciplinary clinical work, real therapeutic decision-making, ethical application in advanced surgery/[27].

## Data Availability

The original contributions presented in this study are included in the article. Further inquiries can be directed to the corresponding author(s).
